# Performance of the 12-lead ECG in predicting short- and long-term risk of sudden cardiac death

**DOI:** 10.1038/s41746-026-02456-1

**Published:** 2026-03-05

**Authors:** Jussi A. Hernesniemi, Teemu Pukkila, Jani Rankinen, Antti Kallonen, Mikko Uimonen, Leo-Pekka Lyytikäinen, Kjell Nikus, Esa Räsänen, Juho Tynkkynen

**Affiliations:** 1 Faculty of Medicine and Health Technology, Tampere University, Tampere, Finland; 2 Heart Hospital, Department of Cardiology, Tampere University Hospital, Tampere, Finland; 3 Computational Physics Laboratory, Faculty of Engineering and Natural Sciences, Tampere University, Tampere, Finland; 4 Centre for Vascular Surgery and Interventional Radiology, Tampere University Hospital, Tampere, Finland

**Keywords:** Cardiology, Computational biology and bioinformatics, Diseases, Medical research

## Abstract

We evaluated the performance of 12-channel ECG in predicting sudden cardiac death across different time intervals using a retrospective data set of 17,625 high-risk cardiac patients who underwent coronary angiography (2007–2018) with follow-up data until 2022. Extreme gradient boosting using 12SL Marquette software-derived parameters from digital ECG recording was used to train and validate models using a random 80/20 split. Model performance was evaluated in both unbalanced and risk-factor-balanced case-control sets. Using single ECG, both long-term (from baseline ECG) and short-term predictions (from the last recorded ECG) achieved a modest area under the curve (AUC) of 0.68 in the unbalanced validation and 0.59/0.63 in the balanced validation (long-/short-term). Adding clinical risk factor data resulted in AUC 0.70/0.71 (unbalanced) and 0.64/0.62 (balanced) for long- and short-term prediction. Adding data of observed ECG changes during follow-up for short-term prediction resulted in the best model performance (0.72/0.66; unbalanced/balanced).

## Introduction

Sudden cardiac deaths (SCD) are thought to account for 20–50% of all CV-deaths^1–3^. Nevertheless, the prediction of SCD beyond left ventricular ejection fraction has proven to be very difficult^4^. The standard 12-lead electrocardiogram (ECG) provides a unique view to the electrical function and properties of the heart^5,6^ and several SCD risk scores have been developed based on traditional risk features^7–9^. Recently, more complex machine learning algorithms have provided promising new approaches to risk stratification^10,11^.

The foremost challenge of traditional ECG risk scores is that they are built on a limited number or risk factors for SCD, such as the presence of left ventricular hypertrophy (LVH) or gross estimates of conduction times, such as QRS duration resulting in low sensitivity for SCD prediction. These factors are also predictors of overall mortality. On the other hand, the challenge of developing complex algorithms, is that the models can be obscure lacking mechanistical insight. In the worst-case scenario, the model may be unintentionally tuned to discriminate unknown features inherent to the testing protocol leading to non-replicable results. Furthermore, most optimistic results have been typically obtained from artificial settings that use only the recent ECG data selected for balanced number of SCD cases and controls^7,11,12^. This type of setting is not possible in real life and lacks proof of applicability in risk prediction in datasets with low incidence of SCD events, long follow-up times and high risk of competing events.

The purpose of this study was to examine the overall performance of standardized 12-channel ECG data in risk prediction and compare the results in different situations using time-to-event data with long and short follow-up times and in a balanced case-control setting. For this end, we used digitally stored and computer-interpreted ECG data with ~500.000 ECGs in a large population of cardiac patients (*n* = 17,585) with applicable follow-up data for SCD events.

## Results

**Fig. 1:**
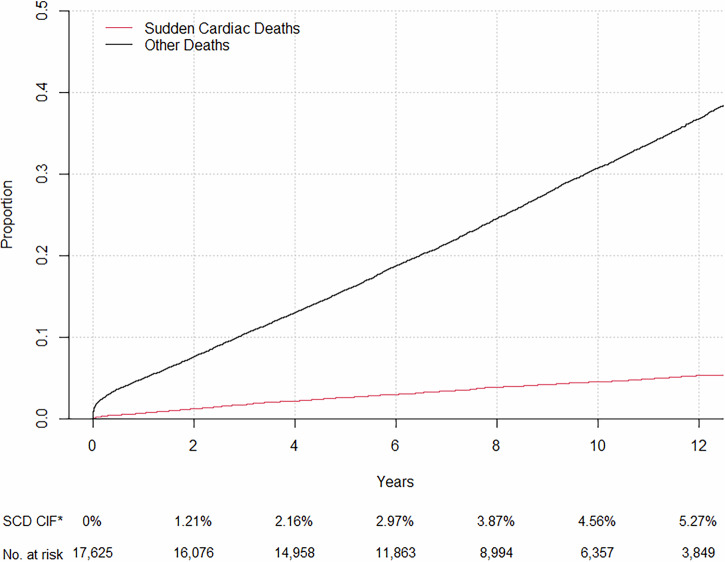
The Cumulative Incidence (CIF) of sudden cardiac deaths and deaths due to other causes during follow-up. The CIF for both sudden cardiac deaths and non-sudden deaths during the course of twelve years among patients in the MADDEC registry who underwent coronary angiography at baseline and then followed for incident deaths.

The general characteristics of the study population (*n* = 17,625) are presented in Table 1. The median follow-up time for all patients was 7.5 years (interquartile range 4.6–10.6 years) during which 832 (4.7%) patients suffered a SCD or SCD equivalent event. The cumulative incidence of SCD over the course of follow-up is depicted in Fig. 1. The mortality due to other causes exceeded 30% during the first ten years of follow-up (Fig. 1).

**Table 1:** General characteristics of the study population

Age at baseline	68 (60–76)
Glomerular Filtration Rate (ml/min/1.73 m^2^)	72.6 (60.3–93.9)
Sex (female)	37.9% (6666)
History of Stroke	7.7% (1354)
Atrial fibrillation or flutter	22.3% (3922)
Hypertension	65.2% (11,471)
Smoking status	
Never	58.5% (10,285)
Past smoker	23.8% (4191)
Active smoker	17.7% (3109)
Previously suffered myocardial infarction	13.6% (2384)
Diabetes (any type)	26.6% (4669)
Peripheral artery disease	6.4% (1127)
Previous coronary artery bypass grafting	8.3% (1450)
Previous percutaneous coronary intervention	8.8% (1554)
Coronary artery disease severity	
No occlusions	31.3% (5504)
1-vessel disease	29.5% (5183)
2-vessel disease	21.4% (3761)
3-vessel disease	17.8% (3137)
Left Main stenosis	7.4% (1301)
Coronary artery disease	71.4% (12,553)
Acute coronary syndrome (as index event)	58.6% (10,314)
Left Ventricular Ejection Fraction	51.4 (45–60)
Killip classification for Heart Failure	
No fluid overload (1)	83.5% (14,678)
Mild congestion (2)	11.3% (1988)
Significant congestion (3)	4.1% (721)
Cardiogenic shock (4)	1.0% (180)
Resuscitated during hospitalization	3.1% (549)
Treatment strategy (after coronary angiography)	
Optimal medical therapy or other intervention	50.3% (8853)
Percutaneous Coronary Intervention	39.3% (6904)
Coronary Artery Bypass Grafting	10.4% (1828)

### Prediction of sudden cardiac death using 12SL parameters and clinical data

**Fig. 2:**
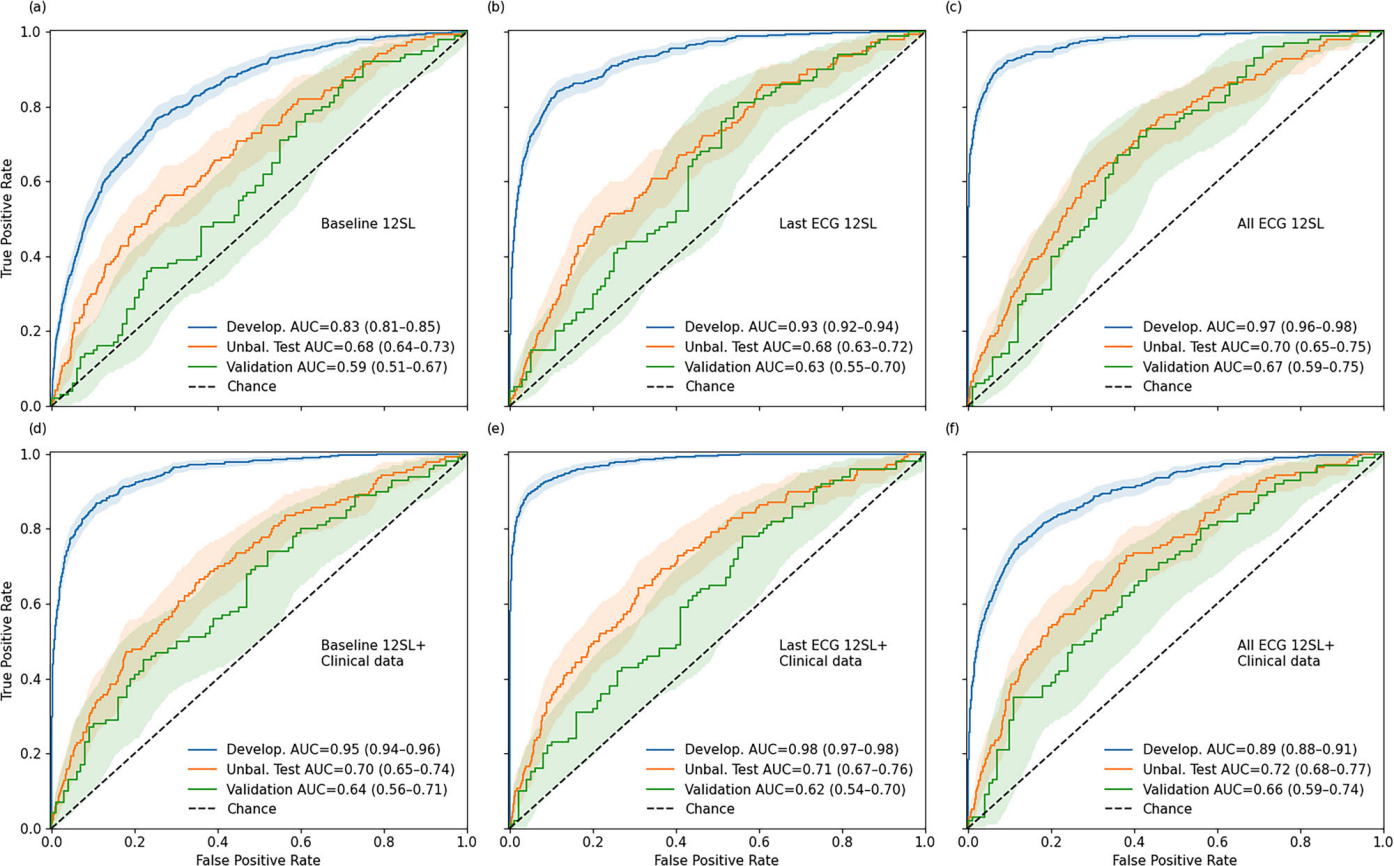
Receiver operating characteristic (ROC) curves (with 95% confidence intervals) illustrating the predictive value of different ECG-based model configurations for incident sudden cardiac death. Area under the curve (AUC) values are shown for the development dataset, the unbalanced validation sample, and the risk factor–balanced validation sample with or without clinical data (top and bottom panels). ROC curves based on ECG parameters measured from baseline (**a**, **d**), from only last recording (**b**, **e**) and from last ECG combined with all previously recorded ECG information (**c**, **f**).

The performance of all risk models with or without clinical risk factors included is presented in Fig. 2. The development models in all three contexts displayed AUC values compatible for probable overfitting with values ranging between 0.83 and 0.98 (Fig. 2). The feature importance metrics for top 100 features in the models are presented in Supplementary Tables 1–3.

**Fig. 3:**
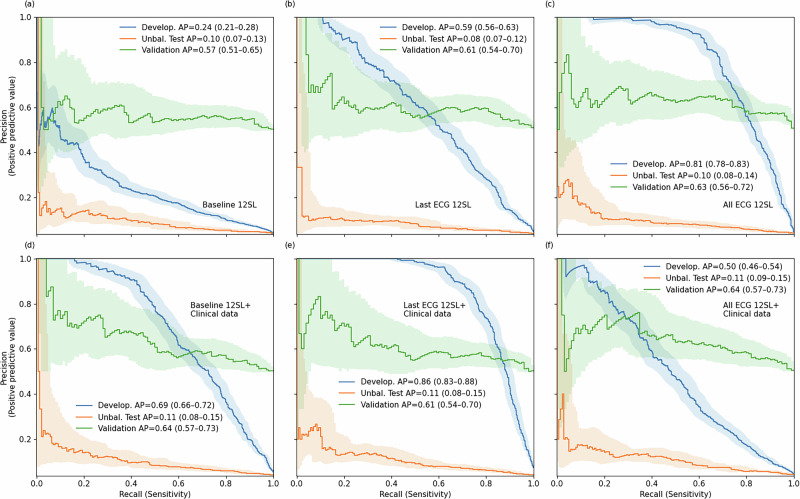
Precision–recall (PRC) curves (with 95% confidence intervals) illustrating the predictive performance of different ECG-based model configurations for incident sudden cardiac death. PRC values are shown for the development dataset, the unbalanced validation sample, and the risk factor–balanced validation sample with or without clinical data (top and bottom panels). ROC curves based on ECG parameters measured from baseline (**a**, **d**), from only last recording (**b**, **e**) and from last ECG combine with all previously recorded ECG information (**c**, **f**).

Evaluating the performance of the developed prediction models in the unbalanced testing set showed that the predictive value of 12SL parameter data resulted in AUC 0.68 for both baseline ECG data to predict long-term risk of SCD and for the last ECG data to predict short-term risk of SCD. Incorporating information from average changes in 12SL parameters observed in previous ECGs did result in a clear improvement to the AUC value (0.70) in short-term prediction for the unbalanced test set and in the balanced validation sample (AUC 0.67). In comparison, the baseline ECG data showed only a very modest AUC for distinguishing between cases and controls in the balanced validation set (AUC 0.59) (Fig. 3.). The model calibrations showed excellent performance for all models in the training data (Brier score of 0.024–0.039) and in the unbalanced training set (0.037–0.038) (Supplementary Fig. 1).

A similar progression in the predictive performance from baseline data to latest ECG data was seen when baseline clinical data (considered variables presented in Table 2) were added to the prediction models with also corresponding improvements in overall performance. In the unbalanced validation clinical data and ECG recorded at baseline (long-term risk) reached AUC of 0.70, whereas clinical data and ECG recorded at last visit reached an AUC of 0.71 for short-term risk. When data from previous ECGs were added to the last ECG and clinical variable the model reached an AUC 0.72 (short-term). The ability of the models to discern between cases and controls in the balanced validation sample reached AUC values of 0.64/0.62/0.66.

**Table 2:** Hyperparameter space for the GridSearch cross-validation

Hyperparameter	Parameter grid
Feature selection	
Correlation_threshold	0.85, 0.95
XGBoost	
N_estimators	50, 100
Max_depth	3, 4, 5
Learning_rate	0.01, 0.05, 0.1
Subsample	0.6, 0.8, 0.9
Lambda	1, 5
Alpha	0.5, 1
Min_child_weight	1, 3

The precision-recall analysis also showed that average precision (AP) did not substantially improve in the unbalanced testing set and balanced validation when using latest ECG data with and without all possible ECG data when compared to baseline (Fig. 3). Overall, the AP values ranged from 0.08 to 0.10. With clinical data added to the prediction models, the AP values reached a value of 0.11. The precision-recall curves the balanced validation sample are not representative of the real-world performance (Fig. 3).

To control for varying short-term follow-up times across the entire study population by differences in when the last ECG was taken, we also performed a sensitivity analysis by limiting the analysis in the short-term prediction to only include ECGs taken within a fixed six-month interval before the end of follow-up (*n* = 7076 available for training, *n* = 1770 available for unbalanced validation). The results were mainly consisted with the results obtained from analysis without a fixed time interval for last ECG (Supplementary Figs. 2, 3).

## Discussion

Using a large retrospective registry study of 17,625 patients with access to over half a million digitally stored ECGs with standardized 12SL parameter ECG data from a period of sixteen years and with applicable follow-up data for SCD events, we made several key observations. First, models based solely on baseline ECG parameters provided only modest predictive value for predicting SCD events in long-term follow-up and poor discriminative ability to discerning between future SCD victims and controls in a balanced validation population. Second, in comparison to baseline ECG data, the predictive performance was better when using the last available ECG prior to the end of follow-up (short-term prediction), illustrating that the closer to the endpoint the ECG data is obtained, the more optimistic the performance metrics appear. Third, adding information from changes in ECG data observed in previous ECGs in addition to the latest ECG data, leads to improved short-term prediction. Fourth, and most importantly, the overall performance of SCD prediction models in a real-world setting seems notably less promising than suggested by earlier case–control studies^7,11,12^.

Overall, our observation of the improvement of the discriminatory value of ECG data when approaching the actual SCD event, are in line with the findings made of the possible utility of repeated ECG risk scoring in SCD risk evaluation in a case-control setting^12^. The foremost problems in estimating the short-term risk prediction (or risk discrimination) value of ECG by selecting the last available ECG either in case-control studies or in retrospective data, is the artificial set up of the analysis. In real-life settings, the true time-to-event data is never known and the true incidence of SCD is low even in traditionally considered high-risk populations^13,14^. While case-control studies have reported AUC values up to 0.82^11^ for only ECG information or even up to AUC 0.88 for ECG and traditional clinical risk factor combined^12^, it is probable that these values are an overestimation of the actual predictive value of ECG data. Supporting this, the results of a very large patient population-based study employing artificial intelligence for model development showed that the predictive ability of ECG data (development set included over one million digitally stored ECG recordings of over 189,000 patients) to predict incident ventricular arrhythmias reached AUC values of 0.76–0.72 in external validation samples of the general population with actual long-term follow-up data^10^.

Overall, our results using preprocessed ECG data for discriminating between SCD victims and controls even in short-term (best AUC of 0.67 in balanced case control setting), fall short from the previous result of the discriminatory value of a deep learning model applied in image analysis of ECGs to discern between SCD cases and controls^11^. Several interesting methodological differences may contribute to the difference. It is possible that using preprocessed data might lead to information loss, that would be amendable by using only raw signal (or image) data for algorithm development. However, as the 12SL algorithm provides a very comprehensive parameter space covering most of the temporal and spatial dimensions of standard 12-channel 10 s ECGs recordings, there are other more plausible explanations for the observed differences. Traditional case-control studies compare cases to controls who are usually still alive and in good clinical condition (or low overall risk of death) when the data is collected. This might yield an overoptimistic assessment of the actual predictive value of any risk classifier because competing events are by default neglected. In our analysis of a high-risk population, the control groups in both unbalanced and balanced validation samples comprised many patients who died of other causes during follow-up with mortality to other causes exceeded 30% over ten years in the entire study population. Furthermore, in the case-control setting of the Oregon SUDS and Ventura PRESTO and many other prospective or retrospective studies of ECG risk factors for SCD, all patients with atrial fibrillation, atrial flutter and paced rhythms were generally excluded^7–9,11,12^. These patients are a major contributor to SCD victims, and their exclusion can ultimately lead to diminished returns in risk prediction. In our study, these patients were included without exception, which means the results are universally applicable at least in patients with CAD who contribute approximately to 70–80% of all SCDs^1^.

While AUC-ROC values provides a measure of overall discrimination, precision–recall analysis offeres a complementary insight into the practical predictive utility of the models, particularly in the context of severe class imbalance. In our unbalanced test sets, where SCD prevalence reflected real-world levels, the AP values were substantially lower than in development or balanced samples, regardless of whether baseline, follow-up, or last ECG data were used. The highest AP value in our data was 0.11. These findings are consistent with prior evidence that precision–recall metrics are more sensitive to class imbalance than ROC analysis. ROC curves may provide overly optimistic impressions of classifier performance in rare event settings, while PRC better reflects real-world utility by focusing on positive predictive value across varying sensitivities^8^. Accordingly, our PRC results underscore the need for caution in interpreting high AUCs in imbalanced data and reinforce the importance of evaluating models using context-appropriate metrics when considering clinical application. However, the PR-AUC values of 0.11 in our long-term risk models correspond to more than a two-fold improvement over the theoretical baseline (0.047, reflecting population event prevalence). This performance gain is consistent with improved discrimination. Whether this would translate into a clinically meaningful improvement in workflow would require a well-defined actionable risk threshold, for example, for guiding ICD therapy. Currently, no such consensus threshold exists.

The results of the present study are based on a cohort of patients undergoing coronary angiography with strict criteria to define SCD and a high autopsy rate, omitting cases with prolonged symptoms, progressive clinical decline, or unclear event descriptions. This population represents patients at elevated risk of SCD, and the results of the modest predictive value for ECG data might be lower in populations with lower SCD incidence and the clinical actionability of ECG-based SCD risk prediction may vary across populations with different cardiac diseases. Disease-specific ECG risk markers may carry substantially different prognostic weight depending on underlying pathology and baseline risk profile. Future work may benefit from population-targeted feature extraction using validated ECG interpretation algorithms such as 12SL, which provide structured, disease-relevant descriptors rather than raw waveform data, supporting more clinically interpretable SCD risk stratification across distinct cohorts.

The XGBoost prediction models employed here were developed as binary risk scores that do not account for unequal follow-up durations across enrollment years. This may lead to individual-level over- or underestimation of SCD risk especially for long-term models. In future model development, the use of a fixed prediction horizon (e.g., 2-year risk) could be a more clinically interpretable alternative, as it also accounts for the effect of mortality due to non-sudden causes to the risk prediction. However, XGBoost as a tree-based gradient boosting algorithm, can capture interaction effects implicitly through recursive splitting of feature space along decision paths providing excellent model performance even using heterogeneous data.

## Methods

This study is based on a retrospective MADDEC registry that comprises information of all patients treated in a single center (The Tampere Heart Hospital) between 2007 and 2018 with follow-up information of serious adverse events updated yearly (follow-up data available until 31.12.2022). Tampere Heart Hospital is the sole service provider of specialized acute cardiac care in the geographically limited area of Pirkanmaa (Finland) with a catchment area of ~500.000 inhabitants. Tampere Heart Hospital also serves as the tertiary center for a larger area of one million inhabitants for cardiothoracic services. Details of the MADDEC registry have been previously published^15,16^.

During the time of the study (2007–2018) altogether 31,188 angiographies were performed on 22,697 patients. For this study, all consecutive patients undergoing their first coronary angiography for acute coronary syndrome (ACS) (*n* = 10.315 or known or suspected coronary artery disease (CAD) in a non-urgent setting (*n* = 12,794) were considered. Patients with unclear indication based on registry data were excluded (*n* = 1523). Patients with suspected ACS who did not receive coronary angiography were excluded due to very poor overall prognosis (~7–8% of all MI patients treated in the Tays Heart Hospital)^17^. After excluding overlapping patients in these cohorts, patients referred to care outside the region of Pirkanmaa (with incomplete follow-up data for ECG) and patients with no available ECG data or incomplete risk factor or demographic data, the final population sample resulted in a sample of *n* = 17,625 patients. The classification of coronary syndromes adhered to the guidelines set forth by the European Society of Cardiology and the American College of Cardiology^18,19^. All basic demographic and procedural data were recorded prospectively to the KARDIO registry by treating physicians. In case of missing information, in-depth revision of all written records and electronic health records was performed. This method has proven to produce reliable information for risk prediction in this patient population^16^.

### Ethics approval

The Finnish legislation stipulates that in retrospective registry studies (such as the MADDEC registry), patient consent is not required, and patients should not be contacted unnecessarily for such a consent. The institutional review board of The Wellbeing Services County of Pirkanmaa approved the study protocol and reaffirming the legislation for not contacting patients for consent (permit R19625). This study adheres to the principles set forth in the Helsinki Declaration on the ethical principles for medical research involving human participants.

### ECG data collection

The ECGs of the patients were collected from the electronic database of the sole service provider for laboratory services in the region of Pirkanmaa (Fimlab Laboratoriot Oy). Specialized healthcare and laboratory diagnostics in Finland, including the region of Pirkanmaa, are highly centralized, leading to almost complete coverage for ECG recordings in these patients between 2007 and 2023. For ECG feature extraction, all ECGs (~500,000) of the included patients recorded between the 1st of January 2007 and the 15th of March 2023 were retrieved from a centralized database (MUSE system) onto a dedicated study database (median of 17 ECGs per patient, with an interquartile range [IQR] of 9–29 ECGs). The GE HealthCare Marquette 12SL algorithm was then used to identify all individual measurable parameters (lead specific wave amplitudes and durations) and subsequently derived features such as PQ interval, QRS duration, QT interval (and heart rate corrected QT intervals by several methods), P wave axis, R wave axis, T wave axis, the presence of atrial fibrillation, atrial flutter, other supraventricular tachycardia and premature ventricular contractions. The measured lead-specific 12SL parameters have been described previously in more detail^20^.

### Follow-up and endpoints

The follow-up period for each patient extended from the initial coronary angiography until true SCD or equivalent event or death by some other mechanism or until December 31st, 2022 for those still alive at the end of the follow-up. The primary endpoint, SCD or equivalent event, comprises all the events that resulted in SCD or would have resulted in SCD unless the event was aborted by external means:

1. Unexpected and sudden natural death from a cardiac cause, occurring within one hour of symptom onset, or in patients found deceased within 24 h of being asymptomatic.
2. An event that would have led to SCD (symptom onset <1 h) but was witnessed and prevented by successful cardiopulmonary resuscitation.
3. Episode of rapid ventricular tachycardia or ventricular fibrillation leading to reliably documented hemodynamic collapse but adequately terminated by implantable cardioverter-defibrillator (ICD) therapy.

Endpoint data were gathered from all written medical records and death certificates, which in Finland have to include a detailed depiction of all circumstances leading to death. The death certificate data is maintained by Statistics Finland, which offers comprehensive data on all deaths among Finnish citizens, and permanent residents, including nearly complete coverage for deaths occurring abroad^21^. As a result, no patients were lost to follow-up in this study (death certificate available for all deceased patients). If the cause of death cannot be determined through clinical information and post-mortem inspection, a medical autopsy is mandated by Finnish law. In cases of sudden or unexpected death, or fatalities occurring within one month of a medical procedure, a medico-legal autopsy is required^22^. A significant proportion of deaths in Finland result in autopsy – 21% of all deaths in 2015 being autopsied^22^ – in the ACS patients of our study population the autopsy rate for all fatal sudden cardiac deaths was 55%^3^. Patients who were successfully resuscitated or who had hemodynamically compromising VF or VT terminated by ICD were identified through a comprehensive screening and review of all medical records at the study center. SCD equivalent cases (resuscitated) were evaluated individually by examination of the depicted timeline of the events and for the primary recorded arrhythmia status. For patients with an ICD device, a systematic review was conducted to determine if they had experienced ventricular arrhythmias. Life-threatening arrhythmias resulting in hemodynamic collapse but successfully managed with ICD therapy, were verified by reviewing patient records, including pacemaker electromyogram (EGM) data, event classification, therapy provided, and event descriptions. When multiple endpoints occurred, the first instance was selected for analysis. If the event timeline (time from symptom onset to hemodynamic collapse) before possible SCD or successfully resuscitated sudden cardiac arrest was unclear or exceeded the one-hour time limit, the event was defined as sudden cardiac arrest (SCA) and was not included as an SCD event. Other deaths of cardiac origin that were clearly non-sudden or expected were classified as other deaths along with deaths due to other causes.

### Statistical analysis

We used three distinct approaches to evaluate value of standardized ECG data in SCD risk prediction:

1. The true predictive value of single recorded ECG data for estimating the long-term risk of SCD (events occurring during follow-up) was analyzed by using 12 SL parameter data extracted from baseline ECG recordings (recorded within a 30-day period after angiography in ACS patients and within 30 days before coronary angiography in other patients).
2. The short-term predictive (or the most optimal discriminatory) value of single ECG data was evaluated by using 12 SL parameter data from the last ECG taken before the end of follow-up (before SCD, before death or last ECG for patients still alive at the end of follow-up) but excluding ECGs that were taken <48 h before death.
3. The short-term predictive (or the most optimal discriminatory) value of all previously accrued ECG data was evaluated by using 12 SL parameter data from the last ECG taken before the end of follow-up (as previously) combined with data of observed changes in each 12SL feature over time in previous ECGs. Given the varying number of measurements at different time points, we calculated the yearly change for each parameter. Specifically, we determined the slope of each parameter in an xy plot, where x represents time in years and y represents the parameter value.

The predictive value ECG information was analyzed by dividing the population into three separate samples for the analysis: Development sample, unbalanced validation sample and balanced validation sample. The results for prediction model performance in each setting are presented in AUC values and by precision-recall curves with average precision (AP) estimates with 95% confidence intervals calculated with bootstrapping, using 2000 iterations and resampling with replacement. First, we extracted a risk factor-matching case-control population (100 SCD cases and 100 controls) to be used in the final validation analysis for any developed prediction model (balanced validation sample). Patients suffering SCA without fulfilling the criteria of SCD were excluded from the controls to reduce any possible confounding caused by possible ambiguity in the endpoint adjudication. Sample was matched for age at baseline, sex, left ventricular ejection fraction and previous coronary artery disease. Matching was performed with propensity score matching^23^ and after the matching effect size measured by Cohen’s d^24^ was below 0.1 for all the matched variables. Second, we divided the remaining population by a random 80%/20% split into development sample (*n* = 13,940, with 588 SCDs) and unbalanced validation sample (*n* = 3485 with 144 SCDs). The resulting development sample was used to train prediction models in each setting. The unbalanced validation sample and the balanced validation sample were used to provide estimates of the true overall long-term and short-term predictive (discriminative) value of ECG data.

### Classification model

**Fig. 4:**
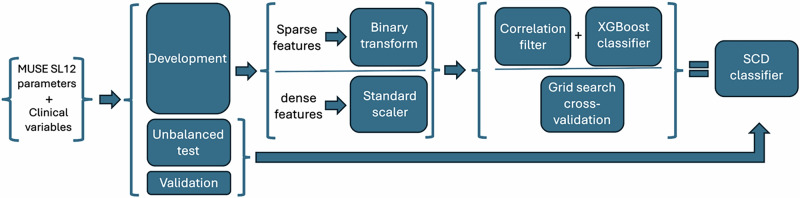
Overview of the classification pipeline, illustrating data preprocessing, feature extraction, model training, and evaluation steps. The classification pipeline shows the steps taken in constructing predictive models in the MADDEC registry using a random 80/20 split of development and validation sets. Extreme gradient boosting classifier was used to train models.

Variable preprocessing and selection were implemented in Python (version 3.8) using *pandas* and *scikit-learn*. Columns with more than 90% zero values were classified as sparse and transformed into binary indicators using a custom function, while remaining dense features were standardized to zero mean and unit variance. Highly correlated features were removed using a custom correlation filter (absolute Pearson correlation >.00.85 or 0.95) to mitigate multicollinearity. Feature selection and model training were performed within a pipeline using XGBoost (*xgboost* package), with hyperparameters optimized via grid search (see Table 2). The overall classification pipeline is illustrated in Fig. 4. Analyses were conducted using R (version 4.3.1; packages *meta*, *cmprsk*) and Python (packages *pandas*, *scikit-learn*, *xgboost*). R was specifically used to estimate cumulative incidence of SCD over the course of follow-up using competing risk methods.

## Supplementary information

ONLINE SUPPLEMENT

## Data Availability

The dataset analysed during the current study is not publicly available due to restrictions imposed by the general data protection regulation and Finnish legislature but the full dataset can be made available for analysis in a fully secure and audited data research environment upon a reasonable request pending the approval of the institutional review board.

## Code availability

The custom code used in this study is not publicly available. The code was developed for research purposes and has not been packaged or documented for external use. As such, it is not currently suitable for public release. Further information may be provided by the corresponding author upon reasonable request.
