# Supplementary material for: Performance of the 12-lead ECG in predicting short- and long-term risk of sudden cardiac death
Source: NPJ Digit Med. 2026 Mar 5;9:317. doi: 10.1038/s41746-026-02456-1 (PMC13079725; doi:10.1038/s41746-026-02456-1)

ONLINE SUPPLEMENT

Predictive Performance of the Standardized 12-Lead ECG in Predicting Short- and Long-Term Risk of Sudden Cardiac Death

Hernesniemi et al.

CONTENT

Supplementary Table 1	Top 100 XGBoost features for baseline model	Pages 2-4
Supplementary Table 2	Top 100 XGBoost features for last ECG model	Pages 5-7
Supplementary Table 3	Top 100 XGBoost features for all ECG model	Pages 8-10
Supplementary Figure 1	Model calibrations	Page 11
Supplementary Figure 2	AUC ROC Curve for limited 6-month predictions	Page 12
Supplementary Figure 3	PR ROC Curve for limited 6-month predictions	Page 13

Supplementary Table 1. Top 100 features sorted by feature importance in the extreme gradient boost model for using baseline model variables (long-term prediction)

FEATURE NAME	IMPORTANCE METRIC
CARDIAC_ARREST_TYPE_DURING_HOSPITALIZATION	0,012573
aVF.Max_S_Ampl	0,011315
aVR.MaxST	0,009602
aVL.TFull_Area	0,007189
aVF.T_PeakAmpl	0,006635
II.S_Area	0,006204
II.SP_Duration	0,006016
V3.P_OnsetAmpl	0,006002
LM_STENOSIS	0,005864
V1.P_OnsetAmpl	0,00568
V1.S_Duration	0,005323
aVR.STM	0,005262
V2.TFull_Area	0,005151
PAST_STROKE	0,005148
PREVALENT_PAD	0,005121
aVF.T_Duration	0,005115
V5.TP_Area	0,005099
aVR.Q_PeakAmpl	0,004999
II.S_PeakTime	0,004794
aVF.QRS_Deflection	0,004684
P_DurationMax	0,00464
V3.STM	0,004579
V3.T_PeakTime	0,00449
II.STE	0,004482
V5.RP_Duration	0,004295
V1.TP_Area	0,004278
V1.T_PeakTime	0,004264
V3.QRS_Balance	0,004228
aVF.S_PeakTime	0,004182
V1.Q_Area	0,004164
aVR.SP_Area	0,004161
CORNPROD	0,004135
III.QRS_Area	0,004131
V5.PFull_Area	0,00409
V6.PP_PeakTime	0,004073
DCM	0,004069
aVF.RP_PeakAmpl	0,004022
V4.T_PeakAmpl	0,00401
V1.TP_Duration	0,00397
V6.SP_Area	0,003882
V6.Q_PeakAmpl	0,003873
aVL.Q_PeakTime	0,003861

V2.S_Area	0,003841
V2.Q_PeakAmpl	0,003753
PREVIOUS_PCI	0,00369
aVF.S_Area	0,003668
V1.STE	0,00366
V2.S_Duration	0,003618
II.R_Area	0,003594
V5.RP_PeakTime	0,003557
aVF.S_PeakAmpl	0,003551
aVL.MinST	0,003548
aVL.P_Duration	0,003524
V1.S_PeakAmpl	0,003524
aVL.PP_PeakAmpl	0,003522
aVR.PP_Duration	0,003518
V2.TP_Duration	0,003517
V1.QRS_Area	0,003501
VFIB_DURING_HOSPITALIZATION	0,003466
V5.SP_Area	0,003428
aVF.T_Special	0,003414
V3.S_Duration	0,003407
V1.S_Area	0,003398
V5.RP_PeakAmpl	0,003389
II.QRS_Area	0,003372
aVF.P_BP	0,003362
V1.Q_PeakTime	0,003349
aVR.PFull_Area	0,003325
aVR.R_PeakAmpl	0,003314
V3.S_PeakAmpl	0,003294
V4.Q_PeakTime	0,003284
V1.Max_R_Ampl	0,003206
II.RP_Duration	0,003202
IAB_class	0,003194
V2.P_Area	0,003179
aVL.P_PeakTime	0,003179
aVF.QRS_Balance	0,003178
II.T_Duration	0,003171
V1.PP_Area	0,003162
aVL.T_PeakTime	0,003154
V1.Q_PeakAmpl	0,003148
V4.PFull_Area	0,003147
II.SP_Area	0,003147
V3.T_End	0,003147
VT_DURING_HOSPITALIZATION	0,003145
V2.T_End	0,003139
V4.PP_Area	0,003125
V4.Max_S_Ampl	0,003122

V6.QRS_Deflection	0,003113
aVR.T_Special	0,003113
V6.T_PeakAmpl	0,003112
V2.STE	0,003072
V2.T_Duration	0,003064
aVL.MaxST	0,003064
V1.PP_PeakTime	0,003007
V3.Max_R_Ampl	0,002991
III.TP_PeakAmpl	0,002985
V5.S_PeakTime	0,002964
V4.SP_PeakTime	0,002958
aVF.T_Area	0,00295

Supplementary Table 2. Top 100 features sorted by feature importance in the extreme gradient boost model using baseline characteristics (clinical information recorded at baseline and ECG data recorded at last visit before the end of follow-up (short-term prediction).

FEATURE	IMPORTANCE METRIC
CARDIAC_ARREST_TYPE_DURING_HOSPITALIZATION	0,012059
aVF.P_BP	0,010981
III.TFull_Area	0,009857
II.TP_PeakTime	0,009341
aVR.MaxST	0,008897
II.RP_PeakTime	0,007811
aVL.R_PeakAmpl	0,00729
III.TP_Duration	0,007279
II.S_PeakAmpl	0,006391
V4.QRS_Balance	0,005762
aVR.PP_PeakAmpl	0,005605
II.S_Duration	0,005594
V2.Q_Area	0,005477
V5.STJ	0,00533
aVF.TP_PeakAmpl	0,005316
II.STM	0,005289
HOITOMUOTO	0,005257
aVL.RP_Area	0,005243
V3.TP_PeakAmpl	0,00518
II.STE	0,005138
II.T_Special	0,005138
aVR.QRS_Deflection	0,005099
aVL.SP_Area	0,004879
V6.TP_Area	0,004843
V4.R_PeakAmpl	0,00475
aVF.TP_Area	0,004723
V1.P_PeakTime	0,00471
V2.TP_Duration	0,004709
LD-V6	0,00467
aVR.TP_Duration	0,004602
MBZI	0,004597
II.TP_PeakAmpl	0,004596
aVR.Q_Area	0,004585
III.PP_Area	0,004526
aVL.STM	0,004436
II.TP_Area	0,004432
LM_STENOSIS	0,004346
V3.Q_Area	0,004283
II.STJ	0,004251
III.T_Special	0,004212

aVF.T_Special	0,004204
aVF.R_PeakTime	0,004168
KILLIP_CLASSIFICATION	0,004148
PREVIOUS_PCI	0,004094
aVR.Q_Duration	0,004045
V6.RP_PeakAmpl	0,00401
PTFV1	0,004009
V2.SP_Area	0,003996
aVL.P_PeakTime	0,003956
V6.SP_PeakTime	0,00394
V3.SP_Duration	0,003866
III.P_PeakAmpl	0,003796
aVR.P_OnsetAmpl	0,003781
V3.R_Duration	0,003775
IRBBB	0,003767
V5.QRS_Balance	0,003736
aVF.MinST	0,00373
aVR.R_Area	0,00373
V3.RP_Area	0,003721
V6.TP_PeakTime	0,003713
II.MinST	0,003709
VFIB_DURING_HOSPITALIZATION	0,003694
II.SP_PeakAmpl	0,003657
aVR.RP_PeakAmpl	0,003647
aVL.SP_PeakAmpl	0,003641
V1.R_PeakTime	0,003632
V3.TP_Duration	0,003627
aVF.PP_PeakAmpl	0,003624
V6.Q_PeakTime	0,003623
V5.P_PeakAmpl	0,003619
V4.T_PeakAmpl	0,003618
V3.S_Area	0,003617
V5.T_Area	0,003588
aVR.T_Special	0,003571
V2.QRSint	0,003563
V1.Max_R_Ampl	0,003541
aVR.PP_PeakTime	0,003541
V1.RP_PeakTime	0,003536
aVL.T_Special	0,003514
aVF.SP_Duration	0,003492
V4.SP_Duration	0,003481
V3.MinST	0,00344
III.RP_Area	0,003429
V4.T_PeakTime	0,003414
PREVALENT_VHD	0,003403
V3.RP_PeakTime	0,003401

V5.SP_Area	0,003384
II.R_PeakTime	0,003368
aVR.P_Duration	0,003351
V1.S_PeakTime	0,00334
V2.T_End	0,003336
aVL.QRSint	0,003335
V6.QRS_Area	0,003332
V6.R_Area	0,003326
PREVALENT_PAD	0,003326
V4.PP_Duration	0,003323
V1.MinST	0,003279
V1.T_PeakAmpl	0,003275
V5.PFull_Area	0,00326
aVL.PP_Area	0,003255

Supplementary Table 3. Top 100 features sorted by feature importance in the extreme gradient boost model using baseline characteristics (clinical information recorded at baseline) and ECG data recorded at last visit before the end of follow-up and using delta-values for ECG parameters (All ECG data + short-term prediction).

FEATURE	**IMPORTANCE METRIC**
SHFT-RGT_slope	0,011783
CARDIAC_ARREST_TYPE_DURING_HOSPITALIZATION	0,011378
V6.Max_S_Ampl_slope	0,011144
ST-(DEC)_slope	0,010947
V6.RP_Duration	0,009532
V6.T_Special	0,008986
QRS11Type_slope	0,008501
AFL_slope	0,008491
V5.P_Duration	0,007704
V3.R_PeakTime	0,007346
V3.STM	0,007279
V5.QRS_Balance	0,006774
V3.TP_Area	0,006636
V3.RP_Area	0,006204
aVL.T_PeakAmpl_slope	0,006203
IAB	0,006065
V3.S_PeakAmpl	0,005894
aVF.Max_R_Ampl_slope	0,005886
V5.STE	0,005766
QUE-CHG_slope	0,005696
V6.Max_S_Ampl	0,005499
aVF.PP_Area_slope	0,005477
NST_slope	0,00544
PREVIOUS_PCI	0,005305
V3.MinST	0,005274
V4.MinST	0,005133
V6.QRS_Deflection	0,005092
V4.RP_PeakAmpl	0,005066
QRS30Time_slope	0,005013
V3.STJ	0,004978
BAE_slope	0,004961
CSEC_slope	0,004881
I.R_PeakAmpl_slope	0,004796
V6.STM	0,004716
SERYR2_slope	0,004702
V3.SP_PeakTime	0,00468
LVH3_slope	0,00461
aVL.P_PeakTime_slope	0,004602
V6.Q_PeakTime_slope	0,004557
V4.SP_Area_slope	0,004536

HOITOMUOTO	0,004534
II.T_PeakAmpl_slope	0,004487
V5.S_PeakTime	0,004465
V3.RP_Area_slope	0,004444
NSTNL_slope	0,004431
V4.TP_Area	0,004393
T-LESINV_slope	0,004321
V2.PP_PeakTime_slope	0,004313
PTFV1.value_slope	0,004254
PMFAIL_slope	0,004252
V3.SP_PeakTime_slope	0,004165
V6.T_PeakAmpl_slope	0,00415
V5.P_PeakAmpl	0,004127
V5.PP_Area	0,004092
V3.STE	0,004087
InjClsInfarction_slope	0,004069
II.QRSint_slope	0,004054
LSBINJ_slope	0,004054
FAV_slope	0,003962
IRBBB	0,003901
V3.QRS_Balance	0,003879
VENT-RAT_slope	0,003878
ALMI_slope	0,003874
QRSType3Count_slope	0,003874
II.P_OnsetAmpl_slope	0,003861
InjClsHypertrophy_slope	0,003848
V3.RP_Duration_slope	0,003843
V4.T_Duration_slope	0,003829
V5.Max_R_Ampl_slope	0,003797
LD-V6	0,003781
aVR.R_Area_slope	0,00378
III.P_BP	0,003777
V5.R_Duration	0,003773
V6.QRSint	0,003745
I.PP_PeakTime_slope	0,003717
V6.MinST	0,00371
V6.RP_PeakTime	0,003705
V2.QRS_Area_slope	0,003691
V6.TFull_Area	0,003681
aVR.RP_Duration_slope	0,003661
OTHER_CMP	0,003659
QRS_GT_120	0,003657
VFIB_DURING_HOSPITALIZATION	0,003634
QRS6Time_slope	0,003626
V3.SP_Area	0,003621
QRS36Type_slope	0,003618

II.T_Area_slope	0,003603
II.S_Area_slope	0,003593
PAxis_slope	0,003583
V5.TP_Duration_slope	0,003581
V5.T_Special_slope	0,00358
V6.P_Area	0,003573
VESC_slope	0,00353
V4.T_End	0,003521
II.T_Duration_slope	0,003515
III.T_PeakAmpl_slope	0,003494
V6.PP_Duration_slope	0,003489
PULD_slope	0,003481
aVL.T_Area_slope	0,003478
V5.TFull_Area	0,003469

Supplementary Figure 1. Model calibration across the entire risk continuum (upper panel) and in the lower range of risk values (lower panel)

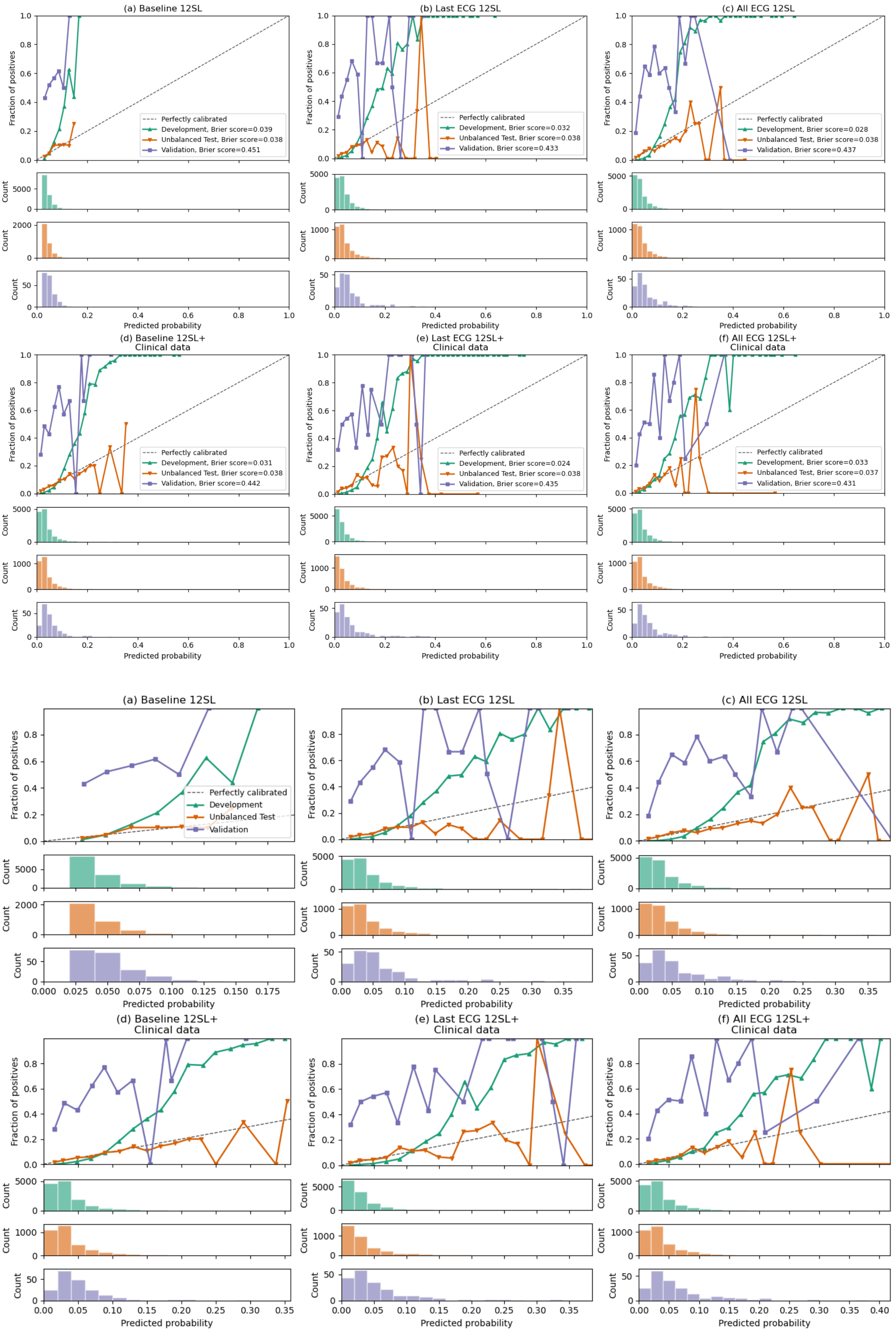

Supplementary Figure 2. Receiver operating characteristic (ROC) curves (with 95% confidence intervals) illustrating the predictive value of different ECG-based model for incident sudden cardiac death with or without clinical data (top and bottom panels). Area under the curve (AUC) values are shown for the development dataset, the unbalanced validation sample, and the risk factor–balanced validation sample. ROC curves are based on ECG parameters measured from only last recording (a & b) taken within six months of the end of the follow-up (n=7076 available for training, n=1770 available for unbalanced validation and 100 cases and 100 controls for balanced validation)

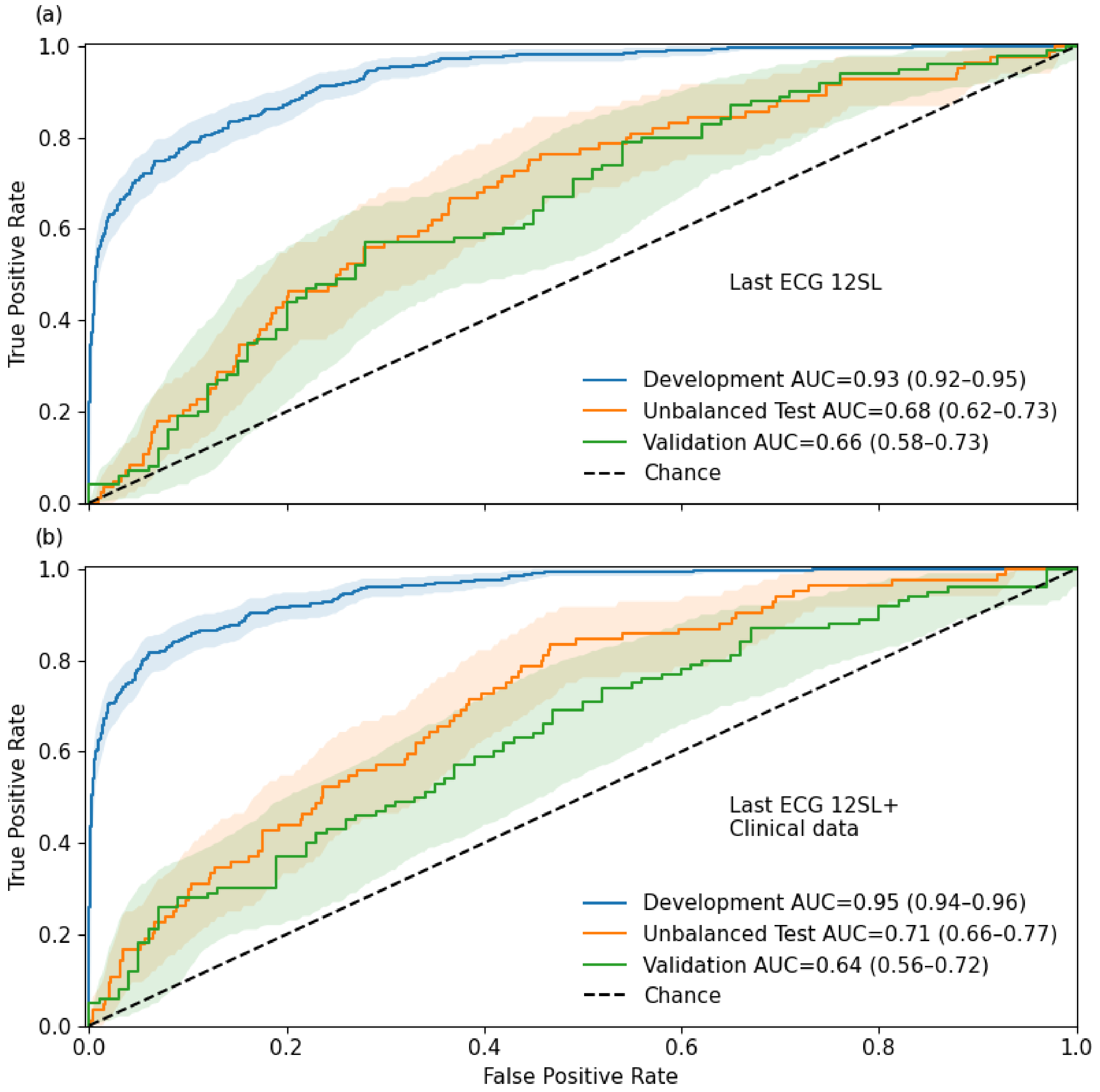

Supplementary Figure 3. Precision–recall (PRC) curves illustrating the predictive performance of different ECG-based model configurations for incident sudden cardiac death. PRC values are shown for the development dataset, the unbalanced validation sample, and the risk factor–balanced validation sample. ROC curves are based on ECG parameters measured from only last recording (a & b) taken within six months of the end of the follow-up (n=7076 available for training, n=1770 available for unbalanced validation and 100 cases and 100 controls for balanced validation)

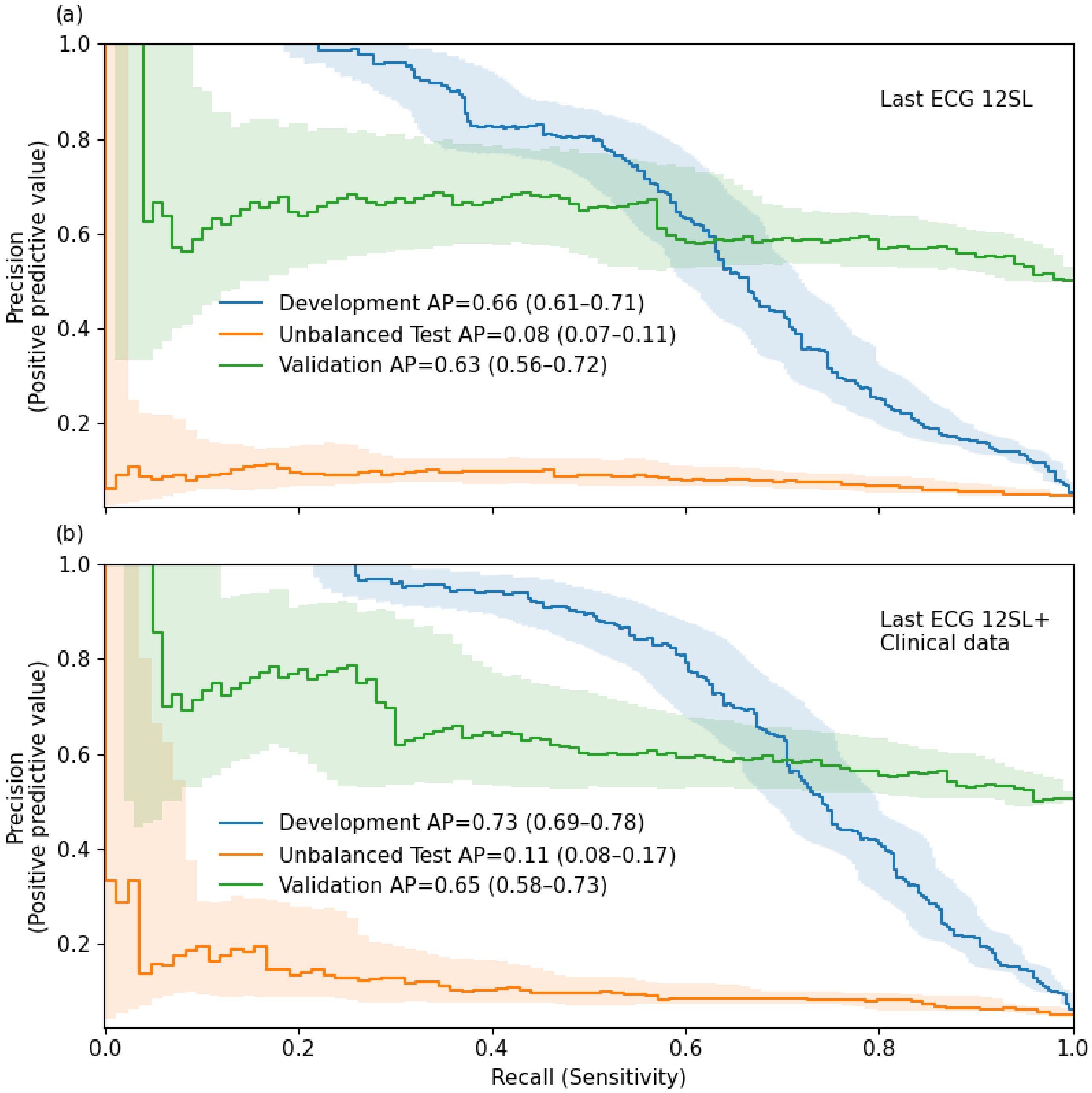

Supplement: Supplementary file 1 — ONLINE SUPPLEMENT [file 41746_2026_2456_MOESM1_ESM.pdf]
